# MAGI3 negatively regulates Wnt/β-catenin signaling and suppresses malignant phenotypes of glioma cells

**DOI:** 10.18632/oncotarget.5323

**Published:** 2015-10-06

**Authors:** Qian Ma, Ying Yang, Duiping Feng, Shuai Zheng, Ran Meng, Pengyan Fa, Chunjuan Zhao, Hua Liu, Ran Song, Tao Tao, Longyan Yang, Jie Dai, Songlin Wang, Wen G. Jiang, Junqi He

**Affiliations:** ^1^ Department of Biochemistry and Molecular Biology, Capital Medical University, Beijing 100069, China; ^2^ Core Facilities Center, Capital Medical University, Beijing 100069, China; ^3^ Department of Interventional Radiology, First Hospital of Shanxi Medical University, Taiyuan 030001, China; ^4^ Department of Pathology, Capital Medical University, Beijing 100069, China; ^5^ Beijing Key Laboratory for Tumor Invasion and Metastasis, Capital Medical University-Cardiff University Joint Centre for Biomedical Research, Cancer Institute of Capital Medical University, Beijing 100069, China; ^6^ Molecular Laboratory for Gene Therapy and Tooth Regeneration, Capital Medical University School of Stomatology, Beijing 100050, China; ^7^ Metastasis and Angiogenesis Research Group, Department of Surgery, Cardiff University School of Medicine, Heath Park, Cardiff, CF14 4XN, U.K

**Keywords:** glioma, β-catenin, MAGI3, PDZ, protein-protein interaction

## Abstract

Gliomas are the most common primary brain malignancies and are associated with a poor prognosis. Here, we showed that the PDZ domain-containing protein membrane-associated guanylate kinase inverted 3 (MAGI3) was downregulated at the both mRNA and protein levels in human glioma samples. MAGI3 inhibited proliferation, migration, and cell cycle progression of glioma cells in its overexpression and knockdown studies. By using GST pull-down and co-immunoprecipitation assays, we found that MAGI3 bound to β-catenin through its PDZ domains and the PDZ-binding motif of β-catenin. MAGI3 overexpression inhibited β-catenin transcriptional activity via its interaction with β-catenin. Consistently, MAGI3 overexpression in glioma cells C6 suppressed expression of β-catenin target genes including Cyclin D1 and Axin2, whereas MAGI3 knockdown in glioma cells U373 and LN229 enhanced their expression. MAGI3 overexpression decreased growth of C6 subcutaneous tumors in mice, and inhibited expression of β-catenin target genes in xenograft tumors. Furthermore, analysis based on the Gene Expression Omnibus (GEO) glioma dataset showed association of MAGI3 expression with overall survival and tumor grade. Finally, we demonstrated negative correlation between MAGI3 expression and activity of Wnt/β-catenin signaling through GSEA of three public glioma datasets and immunohistochemical staining of clinical glioma samples. Taken together, these results identify MAGI3 as a novel tumor suppressor and provide insight into the pathogenesis of glioma.

## INTRODUCTION

Gliomas are the most common primary brain tumors, comprising heterogeneous entities from low-grade to high-grade malignancies. However, nearly all low-grade tumors eventually progress to high-grade malignancies with highly proliferative and invasive phenotypes [1]. Despite multimodal treatment including surgical resection followed by chemo- and radiotherapy, the prognosis of malignant gliomas remains poor and the median survival of patients diagnosed with glioblastoma multiforme (GBM, grade IV glioma) is 9–12 months [2]. Therefore, a better understanding of the mechanisms of gliomagenesis might lead to the identification of novel therapeutic targets or prognostic biomarkers.

Gliomas exhibit a vast array of genetic changes including loss-of-function mutations of the p53 and PTEN tumor suppressors, and hyperactivation of receptor tyrosine kinase signaling [3]. Recently, aberrant activation of Wnt/β-catenin signaling has been implicated in a variety of human cancers including glioma. The Wnt/β-catenin pathway is involved in diverse biological processes such as cell proliferation, survival, differentiation, migration and polarity [4]. In the absence of Wnt stimulation, a β-catenin destruction complex consisting of adenomatous polyposis coli (APC), glycogen synthase kinase 3β (GSK3β) and AXIN phosphorylates β-catenin, resulting in its ubiquitin-mediated proteasomal degradation. On the other hand, the presence of a Wnt ligand causes the disruption of the destruction complex. β-catenin accumulates in the cytoplasm and is translocated to the nucleus, where it binds the transcription factors TCF/LEF to activate its downstream target genes including c-Myc, Cyclin D1, Fra-1 and c-Jun, etc [4, 5]. β-catenin upregulation or activation of its downstream signaling cascades is associated with glioma and positively correlated with tumor grade and poor prognosis [6–12]. Knockdown of β-catenin by small interfering RNA (siRNA) in human glioma cells inhibits cell proliferation, tumorigenicity and invasion [7, 8]. In astrocytomas, β-catenin is responsible for the dysregulation of the reactivation of astrocyte signaling, which plays a key role in the pathogenesis of these tumors [13]. These findings indicate a proto-oncogenic role for β-catenin in gliomagenesis.

Many studies contributed to the elucidation of the molecular mechanisms underlying the regulation of β-catenin signaling, such as destabilization of β-catenin or blockage of its nuclear translocation [5]. An increasing number of PDZ domain-containing proteins have recently emerged as inhibitors of β-catenin transcriptional activity through protein-protein interactions. PDZ domains are conserved modules of approximately 90 amino acids that bind to specific carboxyl-terminal motifs on their target proteins [14]. β-catenin possesses a PDZ binding motif that can bind to several PDZ proteins including TIP-1, DLG, Erbin and NHERF1. Most of these proteins are involved in Wnt/β-catenin-related tumorigenesis such as in colorectal and breast cancers [15–20]. However, a β-catenin interacting PDZ protein involved in the regulation of Wnt signaling and tumor development in glioma has not been fully elucidated to date.

In the present study, we identified a PDZ protein, membrane-associated guanylate kinase inverted 3 (MAGI3) as a novel inhibitor of Wnt/β-catenin signaling and showed that it suppressed the malignant phenotypes of glioma cells. MAGI3 expression was negatively correlated with tumor grade and poor prognosis.

## RESULTS

### MAGI3 expression is downregulated in human glioma

Several lines of evidence suggest that aberrant expression of PDZ proteins is associated with tumorigenesis and/or metastasis [21]. Dysregulation of PDZ protein expression in human glioma has also been reported [22, 23]. To gain insight into the role of PDZ proteins in glioma, we first searched glioma datasets containing normal brain tissues from the Gene Expression Omnibus (GEO) database (http://www.ncbi.nlm.nih.gov/geo/). The gene expression profile of various PDZ proteins in the top 1 largest database GSE4290 was analyzed to identify differentially expressed PDZ genes in human glioma. GSE4290 is a publicly available microarray gene expression dataset comprising 81 glioblastomas and 23 non-tumor brain samples [24]. Among genes encoding PDZ proteins, we found 35 downregulated and 21 upregulated genes in gliomas. Of them, MAGI3 was the most downregulated gene, with 35% reduction of mRNA expression level in gliomas compared with the non-tumor tissues (*P* < 0.01; Supplementary Figure S1A). The similar results were confirmed in two other glioma datasets (GSE7696 and GSE50161) (Supplementary Figure S1B–S1C).

**Figure 1:**
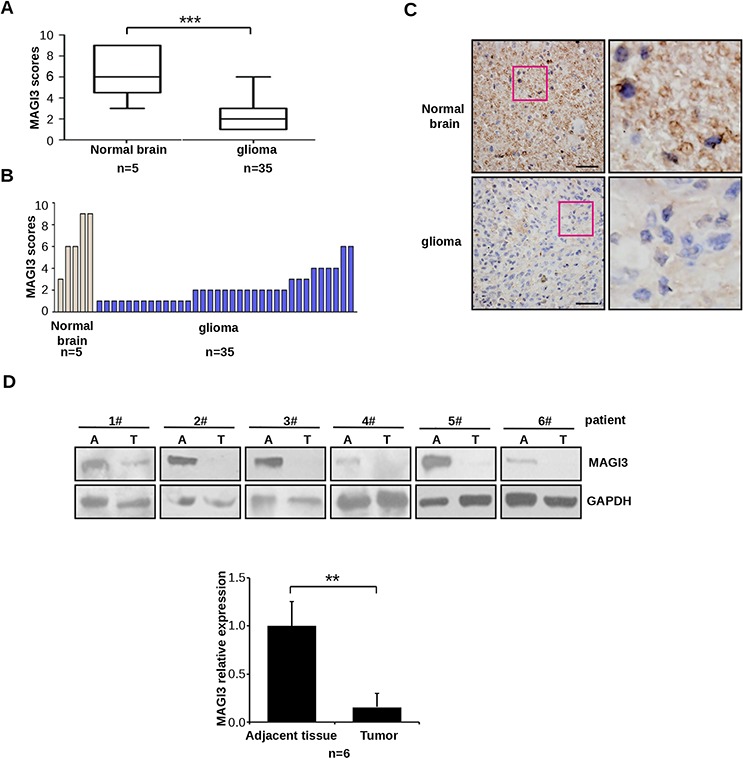
Downregulation of MAGI3 expression in glioma **A–C.** MAGI3 expression was analyzed by immunohistochemistry in a human glioma tissue microarray as described in Materials and Methods. (A) Box plot of relative MAGI3 expression in gliomas and normal brain samples, with the horizontal lines representing the median; the bottom and the top of the boxes representing the 25^th^ and 75^th^ percentiles, respectively; and the vertical bars representing the range of data. Data were analyzed by two-tailed unpaired Student's *t*-test. ***P < 0.001 with respect to the normal population. (B) Plot of individual scores of each glioma and normal brain tissue samples for MAGI3. (C) Representative images from immunohistochemical staining of MAGI3 in tumor and normal brain tissues. The boxed areas in the left images are magnified in the right images. Scale bar represents 40 μm. **D.** MAGI3 expression in 6 independent glioma samples. Top panel: Western blotting result from 6 glioma samples. Bottom panel: The signal intensity of MAGI3 was quantified and normalized to that of GAPDH. Data are expressed as fold change compared to adjacent normal tissues. Results represent the mean ± SD of 6 samples. *P < 0.05 with respect to adjacent tissue.

To verify these findings, MAGI3 expression was examined by immunohistochemistry in a tissue microarray containing 35 human glioma specimens and 5 normal brain tissue samples. MAGI3 protein expression was significantly downregulated in glioma tissues compared with the normal controls (*P* < 0.01; Figure 1A and 1B). Stainings of H&E and astrocyte marker GFAP were also performed on both glioma and normal brain tissue samples (Supplementary Figure S2A–S2B). Taken these results together, MAGI3 was richly expressed in glial cells, whereas only weak or no expression was detected in glioma cells (Figure 1C). The immunohistochemical results were then confirmed by western blotting. As shown in Figure 1D, MAGI3 expression was markedly decreased in glioma tissues from 6 patients, with an approximately 85% reduction in expression levels compared to adjacent normal tissues.

### MAGI3 reduces cell proliferation, causes cell cycle arrest and inhibits glioma cell migration

**Figure 2:**
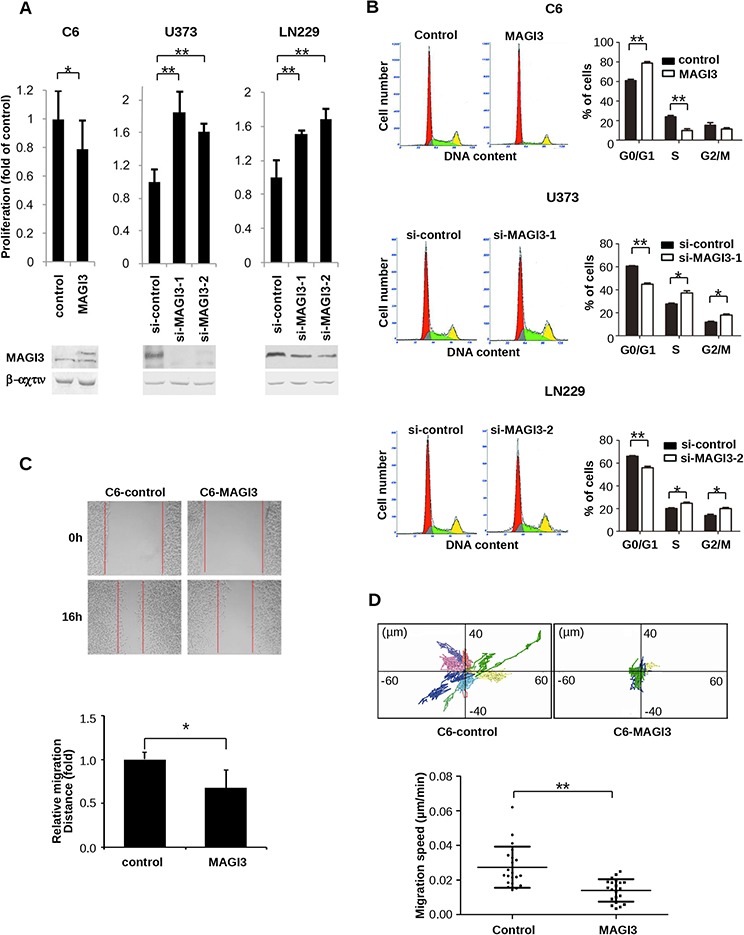
MAGI3 reduces cell proliferation, arrests the cell cycle, and inhibits the migration of glioma cells **A.** Effect of MAGI3 expression on glioma cell proliferation. Control vector or MAGI3-transfected C6 cells and control or MAGI3 siRNA-transfected U373 and LN229 cells were respectively cultured in 96-well plates and stained with CCK8 at 48 h (for C6 cells) or 96 h (for U373 and LN229 cells). Growth of C6 (left panel), U373 (middle panel) and LN229 (right panel) cells was assessed by measuring absorbance at 450 nm with a spectrophotometer. Values are expressed relative to the absorbance in control vector or a scrambled control siRNA-transfected cells. **B.** Effect of MAGI3 expression on cell cycle progression. Cell cycle analysis was performed using propidium iodide DNA staining and flow cytometry. The percentages of C6 (top panel), U373 (middle panel) and LN229 cells (bottom panel) at G0/G1, S, or G2/M phases were determined. Left panel: Representative cell cycle distribution. Right panel: The percentage of cells in different cell cycle phases. **C.** Effect of MAGI3 expression on cell migration in wound-healing assays. Scratch wounds were created in monolayers of control or MAGI3-transfected C6 cells at 0 and 16 h of culture (top panel). The relative migration distance was quantified (bottom panel). (A–C) Data represent the mean ± SD of 3 individual experiments. **D.** Effect of MAGI3 expression on cell migration in two dimensional migration assays. C6 cells transfected with control vector or GFP-MAGI3 were assayed for cell migration. The positions of individual cells were tracked by fluorescence microscopy and recorded every 5 min for 8 h. Top panel: Representative *XY* migration tracks of two different C6 cells. Bottom panel: Analysis of the migration speed of cells. Each group included 21 cells. Data were analyzed by two-tailed paired Student's *t*-test. Error bars represent SD. *P < 0.05, **P < 0.01, with respect to the cells transfected with control vector or siRNA.

To investigate the biologic effects of MAGI3 in glioma, C6 cells, which express moderate levels of endogenous MAGI3, were stably transfected with GFP-MAGI3, whereas U373 and LN229 cells, which express relatively high levels of endogenous MAGI3, were respectively transfected with two different MAGI3 siRNAs. The malignant phenotypes of these glioma cells were then evaluated. MAGI3 overexpression in C6 cells led to decreased cell growth (*P* < 0.01), whereas MAGI3 knockdown in U373 or LN229 cells dramatically promoted cell proliferation (Figure 2A). Assessment of cell cycle progression showed that the proportion of cells in S phase was significantly lower and the number of cells in G0/G1 phase was significantly higher in C6-MAGI3 cells than in control C6 cells. The proportion of cells in G2/M phase was as similar as that in control cells. Conversely, following MAGI3 knockdown in U373 or LN229 cells, the percentage of cells in G0/G1 phase was decreased, whereas the S phase fraction was increased (Figure 2B). Overall, these data indicate that MAGI3 may exert growth inhibitory effects through the induction of G1 arrest.

The effect of MAGI3 on the migration of glioma cells was also assessed using a wound-healing assay. While MAGI3 overexpression significantly inhibited the migration of C6 cells (Figure 2C), MAGI3 knockdown promoted the migration of U373 or LN229 cells (Supplementary Figure S3). To further substantiate these findings, a cell-tracking assay was performed with time-lapse video microscopy, in which the single-cell motility of control C6 and C6-MAGI3 cells was compared. The migration of C6 cells was dramatically suppressed by MAGI3 overexpression (Figure 2D). Taken together, these data indicate that MAGI3 might attenuate glioma cell malignancy.

### MAGI3 interacts directly with β-catenin through its PDZ domains

**Figure 3:**
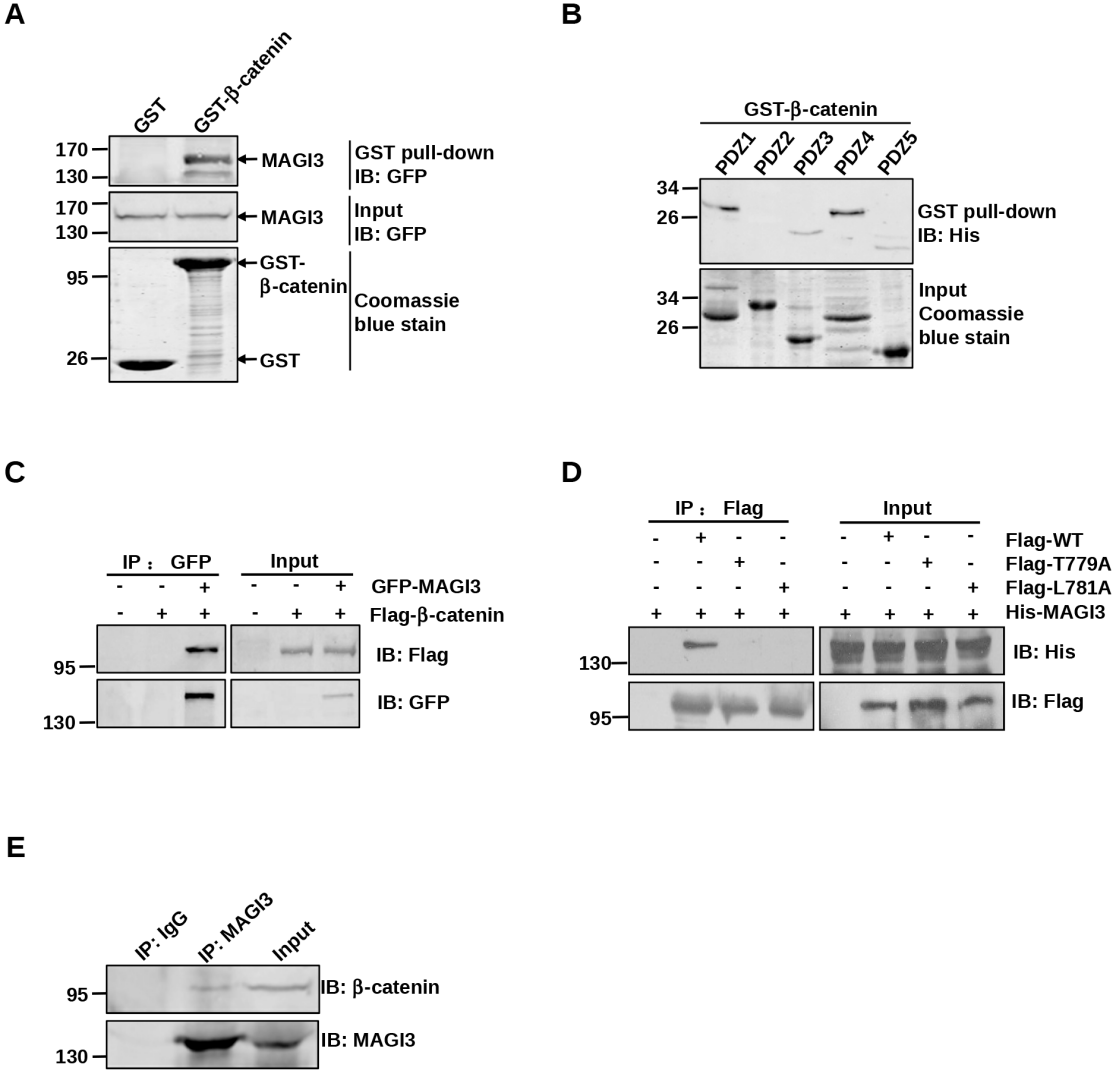
MAGI3 interacts with β-catenin via its PDZ domains and PDZ-binding motif of β-catenin **A.** Association of β-catenin with full-length MAGI3 *in vitro*. GST or GST-β-catenin fusion proteins were used to pull down GFP-tagged full-length MAGI3 from transfected COS-7 cell lysates. The pulled down MAGI3 was detected by western blotting with an anti-GFP antibody (top panel). Coomassie blue staining showed equal loading of the GST fusion proteins (bottom panel). **B.** Binding of β-catenin to multiple PDZ domains of MAGI3. GST-β-catenin fusion proteins were used to pull down His-MAGI3 PDZ1–5 fusion proteins respectively. Precipitates were subjected to western blotting with anti-His antibody (top panel). Coomassie blue staining of His-MAGI3 PDZ fusion proteins used in the pull-down assay (bottom panel). **C.** Interaction of β-catenin with MAGI3 in COS-7 cells. COS-7 cells were transfected with Flag-β-catenin and/or GFP-MAGI3. Cell lysates were immunoprecipitated with anti-GFP antibody, fractionated by SDS-PAGE, and immunoblotted with anti-Flag (top panel) or anti-GFP antibodies (bottom panel). **D.** Interaction of β-catenin with MAGI3 via its PDZ-binding motif. COS-7 cells were transfected with His-MAGI3 and wild-type Flag-β-catenin (WT) or its point mutants (T779A and L781A). Cell lysates were immunoprecipitated using an anti-Flag affinity gel, and immunoblotted with anti-His (top panel) or anti-Flag antibodies (bottom panel). **E.** Binding of MAGI3 to β-catenin in glioma cells. Lysates from C6 cells were subjected to immunoprecipitation with anti-MAGI3 antibody and immunoblotting with anti-β-catenin or anti-MAGI3 antibodies.

β-catenin possesses a PDZ-binding motif at c-terminus that binds to a variety of PDZ domain-containing proteins [15–17, 20], and MAGI3 contains five PDZ domains. To investigate whether MAGI3 may directly associate with β-catenin, GST pull-down assays were performed. As shown in Figure 3A, GST-β-catenin associated robustly with exogenous GFP-MAGI3, whereas GFP-MAGI3 was not detected in the GST control pull-down complex. To identify the specific PDZ domain of MAGI3 responsible for this interaction, each PDZ domain of MAGI3 was subjected to a GST pull-down assay separately. MAGI3-PDZ domains 1, 3, 4 and 5 all bound to GST-β-catenin, with domains 3 and 5 showing weaker binding than domains 1 and 4 (Figure 3B).

To ascertain the cellular interaction between full-length β-catenin and MAGI3, COS-7 cells were co-transfected with Flag-β-catenin and GFP-MAGI3. Immunoprecipitation assay revealed co-immunoprecipitation of the β-catenin/MAGI3 complex (Figure 3C). To examine the structural determinants of the β-catenin/MAGI3 interaction, two of the final four amino acids of β-catenin at positions 0 and −2, the most critical residues for its interaction with PDZ proteins, were mutated separately to alanine (L781A, T779A). COS-7 cells were co-transfected with His-MAGI3 and Flag-tagged wild-type or mutants of β-catenin. As shown in Figure 3D, point mutations completely blocked binding to MAGI3, revealing the requirement of the c-terminus of β-catenin for the β-catenin/MAGI3 interaction. Additionally, coimmunoprecipitation of MAGI3 with β-catenin in C6 cell lysates confirmed their interaction at the endogenous cellular level (Figure 3E). Taken together, these data indicate that the PDZ protein MAGI3 forms a complex with β-catenin *in vitro* and *in cell*.

### MAGI3 represents a novel regulatory element in the Wnt/β-catenin signaling pathway

**Figure 4:**
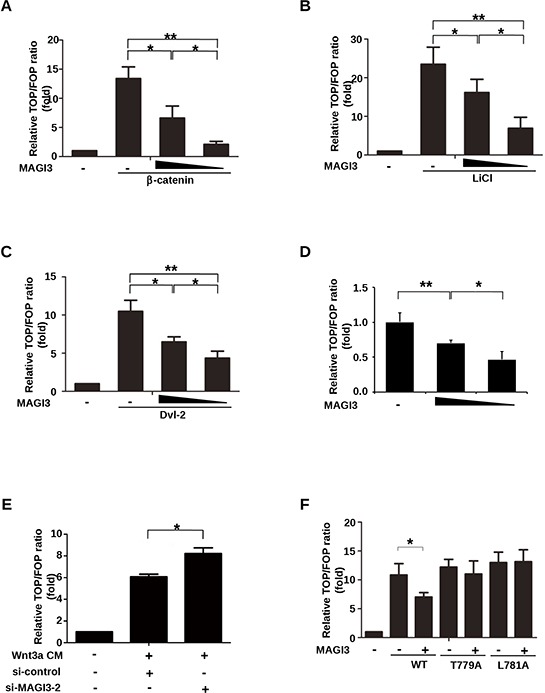
MAGI3 inhibits β-catenin transcriptional activity **A.** HEK293 cells were co-transfected with plasmids coding for β-catenin (0.2 μg), increasing amounts of GFP-MAGI3 (0, 0.1 or 0.2 μg), Renilla (0.01 μg) and either the TOP or FOP luciferase reporter (0.1 μg each). **B.** HEK293 cells were co-transfected with increasing amounts of GFP-MAGI3 (0, 0.2 or 0.4 μg) and luciferase reporter plasmids followed by treatment with 30 mM LiCl for 24 h. **C.** HEK293 cells were co-transfected with Dvl-2 (0.2 μg), increasing amounts of GFP-MAGI3 (0, 0.1 and 0.2 μg) and luciferase reporter plasmids. **D.** HEK293 cells were co-transfected with increasing amounts of GFP-MAGI3 (0, 0.2 and 0.4 μg) and luciferase reporter plasmids. **E.** HEK293 cells were co-transfected with luciferase reporter plasmids and either MAGI3 siRNA or scrambled siRNA control (50 nM each) followed by 8 h of stimulation with Wnt3a CM. **F.** HEK293 cells were co-transfected with GFP-MAGI3 (0.1 μg) and either wild-type β-catenin (WT) or its mutants (T779A, L781A, 0.2 μg each) plus luciferase reporter plasmids. For all transfections, the total amount of DNA was adjusted to 0.5 μg with the empty vector in (A–D) and (F) The reporter activities were determined 48 h after transfection. Values are expressed relative to the ratio of TOP/Renilla versus FOP/Renilla firefly luciferase activity in HEK293 cells transfected with empty vector. Data are expressed as the mean ± SD of triplicate samples. *P < 0.05, **P < 0.01. NS, no significance.

Because PDZ protein TIP-1, Erbin and NHERF1 could repress the transcriptional activity of β-catenin [15, 20, 25], we measured the effect of MAGI3 on the transcriptional activity of β-catenin/TCF using Dual-luciferase Reporter TOP-FOP assay system. HEK293 cells were cotransfected with luciferase reporters and β-catenin in the presence or absence of MAGI3 constructs. MAGI3 inhibited TOP-Flash activity potentiated by β-catenin overexpression in a dose-dependent manner (Figure 4A). In parallel experiments, HEK293 cells were treated with the GSK-3β inhibitor LiCl or transfected with Dvl-2, both of which can stabilize β-catenin in the cytoplasm. MAGI3 overexpression efficiently suppressed the LiCl or Dvl-2-induced increase of β-catenin-TCF activity (Figure 4B–4C). Also, MAGI3 expression repressed endogenous Wnt/β-catenin transcriptional activity in a dose-dependent manner (Figure 4D). Consistently, siRNA-mediated MAGI3 knockdown promoted TOP-Flash activity compared with the scramble control siRNA upon Wnt3a stimulation (Figure 4E). These results indicate that MAGI3 expression retards the activation of the endogenous Wnt/β-catenin pathway.

We next examined whether MAGI3 would also inhibit the transcriptional activity of β-catenin mutants. The c-terminal point mutations of β-catenin without altering the transcriptional activity of β-catenin, abolished the inhibition of TOP-Flash by MAGI3 (Figure 4F), indicating that MAGI3 acts through its interaction with β-catenin.

**Figure 5:**
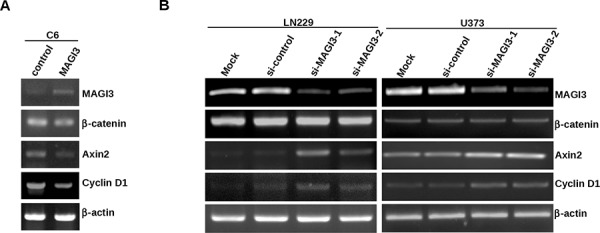
MAGI3 suppresses the expression of target genes of β-catenin signaling in glioma cells **A–B.** Total RNA was isolated from control or MAGI3-transfected C6 cells (A), control or MAGI3 siRNA-transfected U373 or LN229 cells (B) The expressions of MAGI3, β-catenin, Axin2, Cyclin D1 and β-actin mRNA were analyzed by reverse-transcription PCR as described in Materials and Methods. β-actin was used as the internal loading control.

We further assessed the impact of MAGI3 on Wnt/β-catenin downstream target genes in glioma cells. Cyclin D1 is a common target gene of β-catenin pathway, and drives tumorigenesis. Axin2 is a more specific indicator of Wnt/β-catenin signaling. Therefore, expression of both genes has been used as the readouts of the activity of β-catenin signaling in this study. While the mRNA level of β-catenin remained intact, the expression levels of Cyclin D1 and Axin2 were decreased in MAGI3-overexpressing C6 cells (Figure 5A) and increased in MAGI3 knockdown U373 and LN229 cells (Figure 5B). Taken together, these results demonstrate that MAGI3 inhibits Wnt/β-catenin signaling.

### MAGI3 overexpression inhibits the growth of xenograft tumors in nude mice and suppresses the expression of β-catenin target genes in tumors

**Figure 6:**
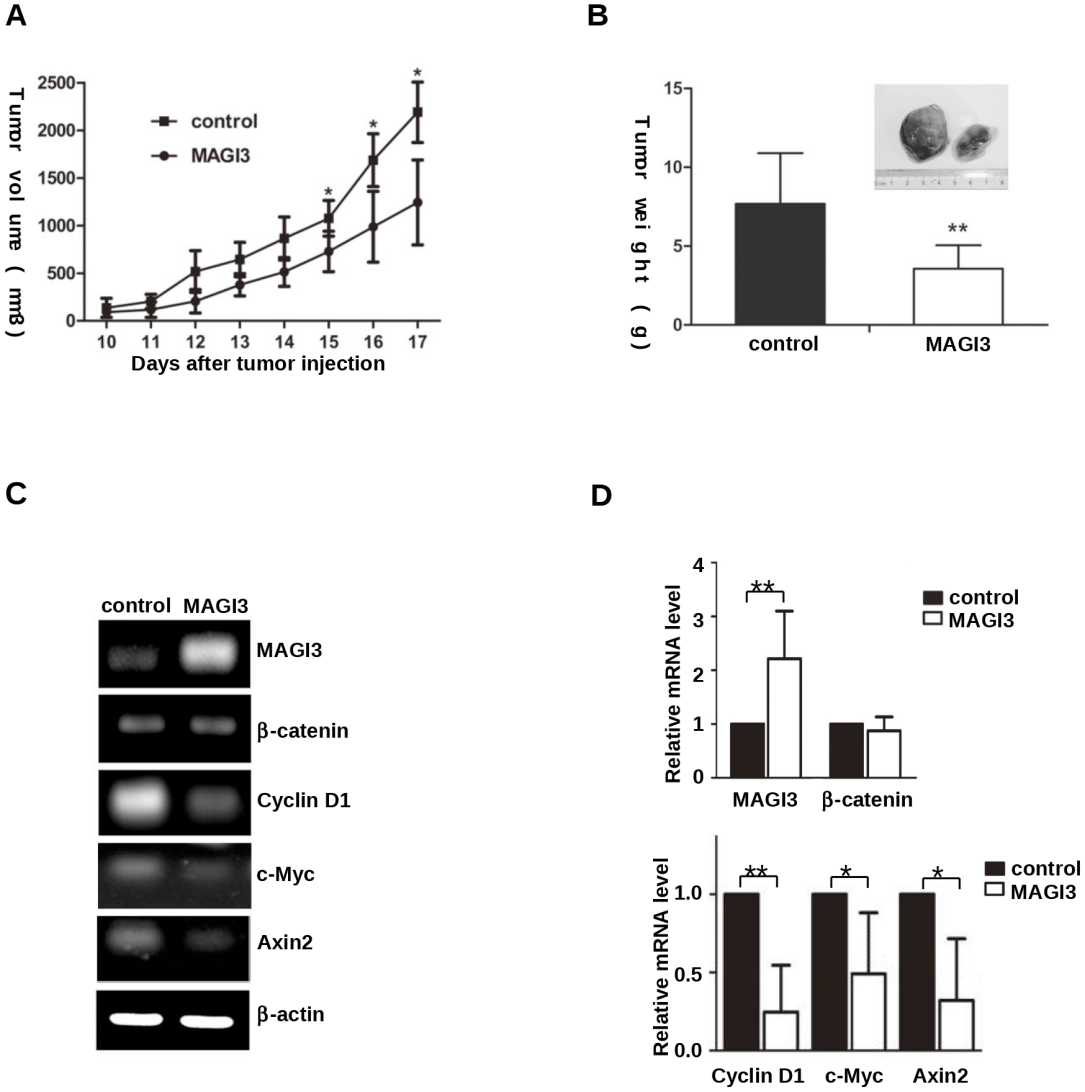
MAGI3 inhibits C6 glioma cell growth and β-catenin target gene expression in xenograft tumors **A.** Growth rate of subcutaneous rat C6 glioma cell tumors. Control or MAGI3-tranfected C6 cells were subcutaneously injected into nude mice. Tumor size was measured daily. Values are expressed as the mean ± SD of 7 tumors in each group. **B.** Weight of the tumors produced by C6 glioma cells. Mice were sacrificed at a minimum of 17 days after transplantation, and xenograft tumors were dissected and individually weighed. **C–D.** Negative correlation between MAGI3 and β-catenin target gene expression in C6 tumors. Total RNA was isolated from dissected tumor samples. The expression of MAGI3, β-catenin, Axin2, c-Myc, Cyclin D1 and β-actin mRNA was analyzed by reverse-transcription PCR. β-actin was used as the internal loading control. (C) Representative image from 7 tumor samples. (D) The expression of the indicated genes in the figure was quantified and expressed as fold change compared to the control. Values are expressed as the mean ± SD of 7 samples. *P < 0.05, **P < 0.01.

To examine the effect of MAGI3 expression *in vivo*, a xenograft tumor model was established by subcutaneous injection of control or MAGI3-overexpressing C6 cells into nude mice. MAGI3 overexpression inhibited the growth of C6 xenografts within 17 days (Figure 6A). Accordingly, the weight of tumors was significantly lower in C6-MAGI3 xenografts than in the control (Figure 6B).

To explore whether MAGI3 suppressed Wnt/β-catenin signaling in xenograft tumors, the expression profiles of MAGI3 and Wnt/β-catenin downstream target genes were examined in primary tumors by semi-quantitative RT-PCR. As shown in Figures 6C and 6D, c-Myc, Cyclin D1 and Axin2 were significantly downregulated in C6-MAGI3 xenografts compared to the controls. In line with the results in glioma cells, MAGI3 did not affect β-catenin mRNA expression in xenograft tumors. The results suggest that MAGI3 may inhibit the growth of C6 glioma xenografts in nude mice by blocking the Wnt/β-catenin pathway.

### MAGI3 expression is correlated with tumor grade, patient survival and activation of Wnt/β-catenin signaling

**Figure 7:**
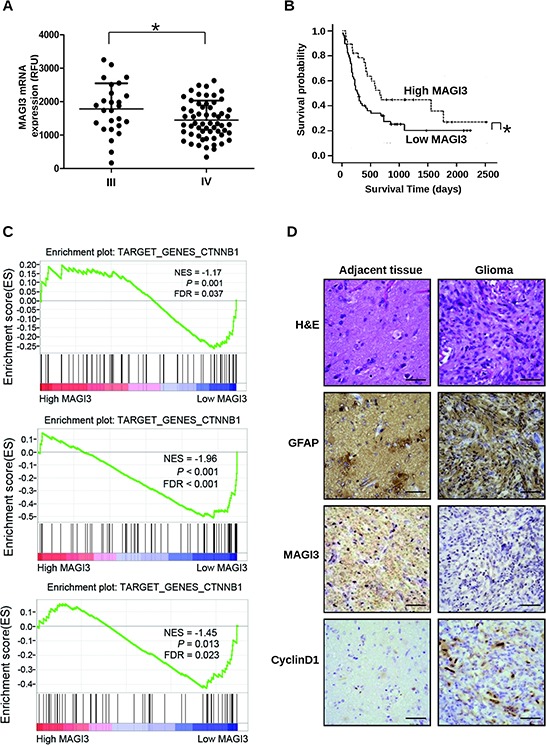
MAGI3 expression is negatively associated with tumor grade, poor prognosis, and activation of Wnt/β-catenin signaling Clinical data and MAGI3 gene expression data of 85 high-grade glioma cases were downloaded from the GEO website (GSE4412). **A.** Scatter plots for MAGI3 mRNA expression analysis in high-grade gliomas. Significance between the 2 populations was determined with a two tailed *t-*test assuming unequal variances. **B.** Kaplan-Meier survival analysis of patients with malignant glioma (*n* = 85) divided into high (greater/equal to median) and low (less than median) MAGI3 expression groups. *Black line*, survival probability of patients with low MAGI3 expression; *dot line*, survival probability of patients with high MAGI3 expression. In (A) and (B), *P < 0.05. **C.** Enrichment plots of gene expression signatures for Wnt/β-catenin pathway according to MAGI3 mRNA expression levels by GSEA of glioma databases GSE4290 (top panel), GSE7696 (middle panel) and GSE50161 (bottom panel). Glioma samples were divided into high and low MAGI3 expression groups. False discovery rate (FDR) gives the estimated probability that a gene set with a given normalized ES (NES) represents a false-positive finding; FDR < 0.05 is a widely accepted cutoff for the identification of biologically significant gene sets. **D.** Glioma and adjacent tissue specimens were analyzed by IHC staining with the indicated antibodies. Scale bar, 400 μm. H&E, hematoxylin and eosin.

To determine the clinical relevance of MAGI3 expression in human glioma, data from a glioma dataset (GEO # GSE4412) containing 85 high-grade gliomas were examined. The results showed that the expression level of MAGI3 was higher in grade III (*n* = 26) than in grade IV gliomas (*n* = 59) (Figure 7A). The data were then divided into high MAGI3 expression and low MAGI3 expression groups, to evaluate the prognosis of these patients. Kaplan-Meier survival analysis showed that high MAGI3 expression group had better outcomes than low MAGI3 expression group in term of survival duration (median survival 810 days vs. 477 days, *P* < 0.05; Figure 7B). The results indicate that MAGI3 might be a prognostic marker for glioma.

To further investigate the correlation between MAGI3 expression and Wnt/β-catenin signaling in glioma, the glioblastoma data in the datasets (GSE4290, GSE7696 and GSE50161) were divided into high MAGI3 expression and low MAGI3 expression groups, and further analyzed using GSEA method. As shown in Figure 7C, the gene set of Wnt/β-catenin target genes, obtained from the Wnt homepage (http://www.stanford.edu/group/nusselab/cgi-bin/wnt/target_genes), was highly enriched in the low MAGI3 expression group (FDR < 0.05). These data suggest a negative correlation between MAGI3 expression and activity of β-catenin signaling in clinical glioma sample. To confirm the relationship of MAGI3 expression and activity of β-catenin signaling in glioma and glial cells, serial sections of glioma and adjacent normal tissue were respectively stained for MAGI3, Cyclin D1 and GFAP by IHC. Similar histological feature was observed in GFAP-, MAGI3- and Cyclin D1-staining sections. GFAP-positive cells were widely distributed in either glioma or adjacent tissues. MAGI3 immunoreactivity was downregulated concomitant with upregulation of Cyclin D1 in glioma cells compared with adjacent normal cells (Figure 7D and Supplementary Figure S4).

## DISCUSSION

The Wnt/β-catenin signaling pathway plays an essential role in regulating the self-renewal, proliferation and differentiation of neural stem cells and progenitor cells in the brain. However, overactivation of β-catenin signaling has been associated with the development of brain tumors including gliomas [26]. Here, we show that the PDZ protein MAGI3 binds to β-catenin, and inhibits its transcriptional activity. MAGI3 is underexpressed in high-grade gliomas compared to normal brain tissues, and MAGI3 overexpression inhibits the malignant transformation of glioma cells. These data support a novel tumor suppressor role of MAGI3 in glioma.

MAGI3 belongs to the MAGUK (membrane-associated guanylate kinase) family, which includes two closely related members, MAGI1 and MAGI2. Similar to the other two family members, MAGI3 contains five PDZ domains (PDZ1–5), one guanylate kinase (GK) domain, and two WW domains [27]. We and others have shown that MAGI3 interacts with a variety of PDZ-binding molecules including β1AR, β2AR, LPA2 and transforming growth factor-α (TGF-α) [28–31]. MAGI3 shares considerable homology with MAGI1 and MAGI2 proteins, which form complexes with β-catenin via their PDZ5 domains [32, 33]. However, unlike them, MAGI3 was shown to physically associate with β-catenin through PDZ1 and PDZ4, as determined by GST pull-down assay (Figure 3B), suggesting differences in the binding properties of MAGI proteins to β-catenin. Similar results were observed for TGF-α, which can bind to the PDZ1 domain of MAGI3, but not to that of MAGI1 [28].

Our study demonstrated that MAGI3 inhibited the transcriptional activity of β-catenin through a direct interaction between the PDZ domains of MAGI3 and the C-terminus of β-catenin. This conclusion is supported by the finding that MAGI3 exerted little effect on the activation of β-catenin mutants, in which MAGI3 binding was impaired (Figures 3D and 4F). In HEK293 cells, MAGI3 interacts with the Frizzled receptors and activates JNK signaling, an alternative Wnt signaling pathway [34]. However, no significant effect of MAGI3 expression on JNK signaling was observed in glioma, as determined by GSEA of the correlation between the expression levels of MAGI3 and genes in the JNK pathway in glioma clinical samples (GEO # GSE4412) (FDR = 0.73, Supplementary Figure S5). These results suggest that the effect of MAGI3 on Wnt/JNK signaling is cell-type specific.

MAGI3 is localized to different subcellular compartments including the cell surface and cytoplasm [35]. Previous studies showed that MAGI3 can recruit PTEN or RPTPβ to plasma membrane via protein-protein interaction and enhance their phosphatase activity [27, 35]. It is possible that MAGI3 could also sequester β-catenin, thereby preventing its nuclear translocation and binding to TCF in the nucleus. In our unpublished study, we found that MAGI3 colocalized with β-catenin predominantly in the cytoplasm by using immunofluorescence assay. MAGI3 expression indeed resulted in cytoplasmic retention of β-catenin, with decreased nuclear localization (unpublished data). These data are consistent with inhibitory effect of MAGI3 on transcriptional activity of β-catenin.

Although the function of MAGIs proteins remains to be defined, their tumor-suppressor potential was indicated by their targeting by viral oncoproteins [36, 37]. Recent research identified MAGI1 as a negative regulator of the Wnt/β-catenin signaling pathway, with tumor-suppressive and anti-metastatic activity in colon cancer [38]. This raises the possibility that MAGI3 might possess a similar ability in human tumors. In the current study, we provide the first evidence that MAGI3 is underexpressed in glioma, and its expression level is negatively correlated with tumor grade and the prognosis of glioma patients (Figures 1 and 7). In glioma cells, MAGI3 overexpression inhibited proliferation and migration, whereas siRNA-mediated knockdown of MAGI3 enhanced cell proliferation (Figure 2). Furthermore, MAGI3 overexpression in C6 glioma cell xenografts suppressed tumor growth *in vivo*. The inhibitory effect of MAGI3 on the malignant behaviors of glioma cells could be attributed to its capacity for suppressing β-catenin signaling in glioma. This hypothesis is supported by the observations: MAGI3 overexpression downregulated the expression of c-Myc and Cyclin D1 (Figures 5 and 6), which are target genes of β-catenin and appear to be associated with glioma progression [39, 40]. Furthermore, although knockdown of MAGI3 expression potentiated cell proliferation in the presence of β-catenin protein expression (Figure 2A), it failed to increase proliferation of β-catenin-knockdown cells (Supplementary Figure S6). These data suggest that MAGI3 inhibits cell proliferation through suppression of β-catenin signaling.

Since MAGI3 is widely distributed in many tissues, it may be involved in the pathogenesis of other tumors besides glioma. Indeed, MAGI3 is downregulated in colon adenocarcinoma tissues and suppresses the LPA-induced malignant phenotypes of colon cancer cells [41]. The results of the present study may provide insight into the tumorigenesis of colon cancer, as MAGI3 downregulation could promote colorectal tumor growth by enhancing β-catenin signaling in colon cancer cells.

The mechanisms that contribute to MAGI3 downregulation in glioma remain to be elucidated. MAGI1 expression in colon cancer cells is downregulated by prostaglandin-E2 (PGE2), a main product of cyclooxgenase-2 (COX-2) [38]. COX-2 is correlated with glioma grade and is a negative prognostic marker in GBMs [42, 43]. In addition, the selective COX-2 inhibitor celecoxib effectively suppresses growth and induces apoptosis in human glioma cells [44]. Given the similarity in structure, subcellular localization and function between MAGI1 and MAGI3, it is tempting to speculate that MAGI3 may also be downregulated by PGE2. Further studies are required to confirm this hypothesis.

In conclusion, the present study identified MAGI3 as a novel tumor suppressor in glioma and showed its inhibitory effect on Wnt/β-catenin signaling. Our findings provide novel clues to improve our understanding of the pathogenesis of glioma.

## MATERIALS AND METHODS

### Antibodies

The anti-His antibody was purchased from MBL (Nagoya, Japan). Anti-MAGI3, anti-Cyclin D1 and anti-GFAP antibodies from Abcam (Cambridge, UK); Anti-β-catenin and anti-GFP antibodies from Cell Signaling Technology (Beverly, CA); Anti-Flag M2 antibody and anti-Flag M2 affinity gels from Sigma (St Louis, MO); Anti-β-actin, anti-GAPDH and HRP-conjugated secondary antibodies from ZSGB-BIO (Beijing, China).

### Glioma patient samples and glioma tissue microarray

Glioma samples were collected from the First Hospital of Shanxi Medical University of Taiyuan during 2010. Patients were included after signing written informed consents. The study was approved by the Ethics Committee of First Hospital of Shanxi Medical University. Diagnosis and classification of glioma were based on the World Health Organization classification. None of the patients included in the study had a family history of glioma or secondary malignancies, and none had received radiotherapy or chemotherapy before surgery. A glioma tissue microarray, containing biopsies from 35 different gliomas and 5 normal human brain specimens was obtained from US Biomax (Rockville, MD).

### Immunohistochemistry

Glioma and adjacent normal tissues were fixed with 4% formalin, prepared as paraffin-embedded section. Immunohistochemistry was performed as described previously [45]. For tissue microarray, each specimen was analyzed in duplicate. The density of MAGI3 staining in each specimen was scored according to the percentage of positively staining cells counted in 20 fields at 200× magnification and the scores were as follows: 1 for <25%; 2 for 25–50%; 3 for >75%. The intensity of MAGI3 immunostaining was scored as 0, 1, 2, and 3 for no, weak, moderate, and strong immunoreactivity, respectively. The density scores were multiplied by the corresponding immunostaining intensity scores to obtain histological scores of MAGI3 expression.

### RNA extraction and RT-PCR

Total RNA was isolated using the Trizol reagent (Invitrogen, Carlsbad, CA) and reverse transcription was performed with a Protoscript M-Mulv First strand cDNA Synthesis Kit (New England Biolabs, Beverly, MA). The cDNAs obtained were mixed with polymerase chain reaction (PCR) solution containing the primers indicated in Supplementary Table S1. The following PCR was cycled 35 times at 94°C for 45 s, 55°C for 45 s, and 72°C for 45 s. The PCR products were resolved by agarose gels. Gene expression was normalized against β-actin in the same sample.

### Cell lines

Human or rat glioblastoma cell lines (U373 and C6), the HEK293 and COS-7 cell lines were from the European Collection for Animal Cell Culture (ECACC, Porton Down, Salisbury, UK). The human glioblastoma cell line LN229 was purchased from American Type Culture Collection (ATCC, Manassas, VA). Parental and Wnt3a-expressing L cells were kindly provided by Dr. Clever Hans (KNAW and University Medical Center Utrecht, Uppsalaan, the Netherlands). All cells were maintained in DMEM supplemented with 10% fetal bovine serum (FBS) and grown at 37°C in 5% CO_2_ at constant humidity. The Wnt3a-conditioned medium (CM) and L cells control CM were prepared as described previously [46].

### Plasmids, siRNA and transfection

The constructs encoding Flag-tagged β-catenin, Renilla luciferase, TOP-Flash or FOP-Flash luciferase reporters and the pGEX-4T-1-β-catenin vector were kindly provided by Dr. Wei Wu (Tsinghua University, Beijing, China). Point mutations of β-catenin were introduced into the Flag-β-catenin construct by PCR and verified by bidirectional sequencing. The plasmid for Myc-tagged Dvl-2 was kind gift from Dr. Yeguang Chen (Tsinghua University, Beijing, China), and pEGFP-N4-MAGI3 and pcDNA3-V5/His-MAGI3 were kindly provided by Dr. Randy Hall (Emory University, GA). pET30A plasmids containing the PDZ domains of MAGI3 were generated as described previously [29].

SiRNAs and their sources were as follows: MAGI3 siRNA-1 targeted the sequence 5′-GGUCCACCAUCAGGAACAAACUCAG-3′; MAGI3 siRNA-2 targeted the sequence 5′-GAGAUGGACC UGACCAGUCUAUAUA-3′ (Invitrogen, Carlsbad, CA). β-catenin siRNA (sc-29209) was obtained from Santa Cruz Biotechnology. The control siRNA (si-control) was 5′-UUCUCCGAACGUGUCACGUTT-3′ (Invitrogen).

Cell transfection was performed using Lipofectamine 2000 (Invitrogen). For siRNA knockdown and transient transfection experiments, RNA or protein extraction was performed 48 h post-transfection. For establishment of stable GFP-MAGI3-expressing cells, C6 cells were transfected with constructs of pEGFP-N4-MAGI3. At 24 h after transfection, the cells were selected with 1 mg/mL G418 (Calbiochem, San Diego, CA) in culture medium for an additional 14 days. Expression of the GFP-MAGI3 protein was verified by western blotting.

### TOP/FOP luciferase assay

The assay was performed as described previously [38]. Luminescence was measured with an EnVision 2104 multilabel reader (PerkinElmer, Waltham, MA). In some experiments, the cells were pretreated with 30 mM LiCl for 24 h or Wnt3a-enriched medium for 8 h.

### Cell proliferation assay

Cells were cultured in 96-well microplates at a density of 3000 per well. MAGI3-overexpressing or siRNA-transfected cells were seeded in DMEM medium containing 5% FBS 24 h post-transfection. At 48 h (C6 cells) or 96 h (U373 and LN229 cells) after plating, CCK-8 (Dojindo, Kumamoto, Japan) was added. Viable cells were quantified by measuring absorbance at 450 nm with an EnVision 2104 multilabel reader (PerkinElmer, Waltham, MA).

### Wound-healing assay

Control and stable GFP-MAGI3-transfected C6 cells were cultured at a density of 5 × 10^5^ cells/well in a 6-well plate and allowed to reach confluence. The cell monolayer was carefully scratched using a sterile 200 μL pipette tip across the center of the well. Images of the wound were recorded under a phase contrast microscope at 0 and 16 h after culture. The widths of three different wound surfaces in each group were noted and measured using Image J analysis software.

### Live-cell time-lapse recording for cell tracking

Control and stable GFP-MAGI3-expressing C6 cells were seeded into an eight-chambered cover glass (Lab-Tek Chambered NO 1.0 Borosilicate Cover Glass System, NY) and cultured in DMEM. The random migration of the cells was recorded every 5 min for 8 h under an inverted fluorescence microscope (Olympus, Tokyo, Japan) with the confocal imaging system Ultra VIEW Vox (PerkinElmer, Waltham, MA). The traces and migration speed of cells were analyzed with the velocity as described previously [45].

### GST pull-down, co-immunoprecipitation and western blotting

Purification of GST- or His-tagged proteins and GST pull-down assay, lysis of cells and tumor samples, co-immunoprecipitation and western blotting were performed as described previously [29].

### Cell cycle analysis

Cells in the log phase of growth were harvested and fixed with 70% ethanol overnight at 4°C. The cells were then incubated with 0.1% Triton X-100 solution containing 50 μg/mL propidium iodide (Sigma, St Louis, MO) and RNase in the dark for 1 h at room temperature. A total of 1 × 10^5^ cells were analyzed for their DNA content using an EPICS@XL flow cytometer (Beckman Coulter, Brea, CA). Data acquisition was performed with EXPO32 ADCv1.1C MultiCycle software (Beckman Coulter, Brea, CA).

### *In vivo* tumorigenicity assay

All animal experiments were performed following the National Institutes of Health Guide for the Care and Use of Laboratory Animals and were approved by the Animal Use and Care Committee of Capital Medical University. Control or stable GFP-MAGI3-expressing C6 cells (1 × 10^5^) in 0.1 mL PBS were subcutaneously injected into the right flank of male Balb/c nude mice (4–5 weeks). Each group included 7 mice. Tumor size was measured daily with calipers and tumor volume was estimated using the following formula: volume = (length × width^2^)/2. The mice were sacrificed on day 17 post-injection, and tumors were harvested and individually weighed. Following resection, a portion of each tumor sample was prepared for isolation of RNA.

### Gene set enrichment analysis

The association between expression of MAGI3 and biological processes was analyzed using Gene Set Enrichment Analysis (GSEA v2.0,http://www.broad.mit.edu/gsea/). GSEA calculates a pathway Enrichment Score (ES) that estimates whether genes from pre-defined gene set of Wnt/β-catenin target genes (http://www.stanford.edu/group/nusselab/cgi-bin/wnt/target_genes) are enriched among the highest- (or lowest-) ranked genes or distributed randomly. Default settings were used. Thresholds for significance were determined by permutation analysis (1000 permutations). False Discovery Rate (FDR) was calculated. A gene set is considered significantly enriched when the FDR score is <0.05.

### Statistical analysis

Statistical analyses were performed using the SPSS 18.0 (SPSS Inc, Chicago, IL) and Graphpad Prism 5 (Graphpad software Inc, San Diego, CA). Group distributions were compared using the Student's *t* test or one-way analysis of variance. A value of *P* < 0.05 was considered statistically significant.

## SUPPLEMENTARY FIGURES AND TABLE



## References

[1] Louis DN (2006). Molecular pathology of malignant gliomas. Annu Rev Pathol.

[2] Umesh S, Tandon A, Santosh V, Anandh B, Sampath S, Chandramouli BA, Sastry Kolluri VR (2009). Clinical and immunohistochemical prognostic factors in adult glioblastoma patients. Clin Neuropathol.

[3] Nager M, Bhardwaj D, Canti C, Medina L, Nogues P, Herreros J (2012). beta-Catenin Signalling in Glioblastoma Multiforme and Glioma-Initiating Cells. Chemother Res Pract.

[4] Anastas JN, Moon RT (2013). WNT signalling pathways as therapeutic targets in cancer. Nat Rev Cancer.

[5] Clevers H, Nusse R (2012). Wnt/beta-catenin signaling and disease. Cell.

[6] Liu C, Tu Y, Sun X, Jiang J, Jin X, Bo X, Li Z, Bian A, Wang X, Liu D, Wang Z, Ding L (2011). Wnt/beta-Catenin pathway in human glioma: expression pattern and clinical/prognostic correlations. Clin Exp Med.

[7] Liu X, Wang L, Zhao S, Ji X, Luo Y, Ling F (2011). beta-Catenin overexpression in malignant glioma and its role in proliferation and apoptosis in glioblastma cells. Med Oncol.

[8] Pu P, Zhang Z, Kang C, Jiang R, Jia Z, Wang G, Jiang H (2009). Downregulation of Wnt2 and beta-catenin by siRNA suppresses malignant glioma cell growth. Cancer Gene Ther.

[9] Sareddy GR, Panigrahi M, Challa S, Mahadevan A, Babu PP (2009). Activation of Wnt/beta-catenin/Tcf signaling pathway in human astrocytomas. Neurochem Int.

[10] Utsuki S, Sato Y, Oka H, Tsuchiya B, Suzuki S, Fujii K (2002). Relationship between the expression of E-, N-cadherins and beta-catenin and tumor grade in astrocytomas. J Neurooncol.

[11] Rossi M, Magnoni L, Miracco C, Mori E, Tosi P, Pirtoli L, Tini P, Oliveri G, Cosci E, Bakker A (2011). beta-catenin and Gli1 are prognostic markers in glioblastoma. Cancer Biol Ther.

[12] Wu W, Tian Y, Wan H, Ma J, Song Y, Wang Y, Zhang L (2013). Expression of beta-catenin and E- and N-cadherin in human brainstem gliomas and clinicopathological correlations. Int J Neurosci.

[13] Yang C, Iyer RR, Yu AC, Yong RL, Park DM, Weil RJ, Ikejiri B, Brady RO, Lonser RR, Zhuang Z (2012). beta-Catenin signaling initiates the activation of astrocytes and its dysregulation contributes to the pathogenesis of astrocytomas. Proc Natl Acad Sci USA.

[14] Fanning AS, Anderson JM (1999). PDZ domains: fundamental building blocks in the organization of protein complexes at the plasma membrane. J Clin Invest.

[15] Kanamori M, Sandy P, Marzinotto S, Benetti R, Kai C, Hayashizaki Y, Schneider C, Suzuki H (2003). The PDZ protein tax-interacting protein-1 inhibits beta-catenin transcriptional activity and growth of colorectal cancer cells. J Biol Chem.

[16] Subbaiah VK, Narayan N, Massimi P, Banks L (2012). Regulation of the DLG tumor suppressor by beta-catenin. Int J Cancer.

[17] Kreimann EL, Morales FC, de Orbeta-Cruz J, Takahashi Y, Adams H, Liu TJ, McCrea PD, Georgescu MM (2007). Cortical stabilization of beta-catenin contributes to NHERF1/EBP50 tumor suppressor function. Oncogene.

[18] Liu D, Shi M, Duan C, Chen H, Hu Y, Yang Z, Duan H, Guo N (2013). Downregulation of Erbin in Her2-overexpressing breast cancer cells promotes cell migration and induces trastuzumab resistance. Mol Immunol.

[19] Pan Y, Wang L, Dai JL (2006). Suppression of breast cancer cell growth by Na+/H+ exchanger regulatory factor 1. Breast Cancer Res.

[20] Ress A, Moelling K (2008). The PDZ protein erbin modulates beta-catenin-dependent transcription. Eur Surg Res.

[21] Subbaiah VK, Kranjec C, Thomas M, Banks L (2011). PDZ domains: the building blocks regulating tumorigenesis. Biochem J.

[22] Chen J, Xu J, Zhao W, Hu G, Cheng H, Kang Y, Xie Y, Lu Y (2005). Characterization of human LNX, a novel ligand of Numb protein X that is downregulated in human gliomas. Int J Biochem Cell Biol.

[23] Wang H, Han M, Whetsell W, Wang J, Rich J, Hallahan D, Han Z (2014). Tax-interacting protein 1 coordinates the spatiotemporal activation of Rho GTPases and regulates the infiltrative growth of human glioblastoma. Oncogene.

[24] Sun L, Hui AM, Su Q, Vortmeyer A, Kotliarov Y, Pastorino S, Passaniti A, Menon J, Walling J, Bailey R, Rosenblum M, Mikkelsen T, Fine HA (2006). Neuronal and glioma-derived stem cell factor induces angiogenesis within the brain. Cancer Cell.

[25] Wheeler DS, Barrick SR, Grubisha MJ, Brufsky AM, Friedman PA, Romero G (2011). Direct interaction between NHERF1 and Frizzled regulates beta-catenin signaling. Oncogene.

[26] Gong A, Huang S (2012). FoxM1 and Wnt/beta-catenin signaling in glioma stem cells. Cancer Res.

[27] Wu Y, Dowbenko D, Spencer S, Laura R, Lee J, Gu Q, Lasky LA (2000). Interaction of the tumor suppressor PTEN/MMAC with a PDZ domain of MAGI3, a novel membrane-associated guanylate kinase. J Biol Chem.

[28] Franklin JL, Yoshiura K, Dempsey PJ, Bogatcheva G, Jeyakumar L, Meise KS, Pealsall RS, Threadgill D, Coffey RJ (2005). Identification of MAGI-3 as a transforming growth factor-alpha tail binding protein. Exp Cell Res.

[29] He J, Bellini M, Inuzuka H, Xu J, Xiong Y, Yang X, Castleberry AM, Hall RA (2006). Proteomic analysis of beta1-adrenergic receptor interactions with PDZ scaffold proteins. J Biol Chem.

[30] Yang X, Zheng J, Xiong Y, Shen H, Sun L, Huang Y, Sun C, Li Y, He J (2010). Beta-2 adrenergic receptor mediated ERK activation is regulated by interaction with MAGI-3. FEBS Lett.

[31] Zhang H, Wang D, Sun H, Hall RA, Yun CC (2007). MAGI-3 regulates LPA-induced activation of Erk and RhoA. Cell Signal.

[32] Dobrosotskaya IY, James GL (2000). MAGI-1 interacts with beta-catenin and is associated with cell-cell adhesion structures. Biochem Biophys Res Commun.

[33] Nishimura W, Yao I, Iida J, Tanaka N, Hata Y (2002). Interaction of synaptic scaffolding molecule and Beta-catenin. J Neurosci.

[34] Yao R, Natsume Y, Noda T (2004). MAGI-3 is involved in the regulation of the JNK signaling pathway as a scaffold protein for frizzled and Ltap. Oncogene.

[35] Adamsky K, Arnold K, Sabanay H, Peles E (2003). Junctional protein MAGI-3 interacts with receptor tyrosine phosphatase beta (RPTP beta) and tyrosine-phosphorylated proteins. J Cell Sci.

[36] Thomas M, Glaunsinger B, Pim D, Javier R, Banks L (2001). HPV E6 and MAGUK protein interactions: determination of the molecular basis for specific protein recognition and degradation. Oncogene.

[37] Thomas M, Laura R, Hepner K, Guccione E, Sawyers C, Lasky L, Banks L (2002). Oncogenic human papillomavirus E6 proteins target the MAGI-2 and MAGI-3 proteins for degradation. Oncogene.

[38] Zaric J, Joseph JM, Tercier S, Sengstag T, Ponsonnet L, Delorenzi M, Ruegg C (2012). Identification of MAGI1 as a tumor-suppressor protein induced by cyclooxygenase-2 inhibitors in colorectal cancer cells. Oncogene.

[39] Wang J, Wang H, Li Z, Wu Q, Lathia JD, McLendon R E, Hjelmeland AB (2008). c-Myc is required for maintenance of glioma cancer stem cells. PLoS One.

[40] Wang J, Wang Q, Cui Y, Liu ZY, Zhao W, Wang CL, Dong Y, Hou L, Hu G, Luo C, Chen J, Lu Y (2012). Knockdown of cyclin D1 inhibits proliferation, induces apoptosis, and attenuates the invasive capacity of human glioblastoma cells. J Neurooncol.

[41] Lee SJ, Ritter SL, Zhang H, Shim H, Hall RA, Yun CC (2011). MAGI-3 competes with NHERF-2 to negatively regulate LPA2 receptor signaling in colon cancer cells. Gastroenterology.

[42] Joki T, Heese O, Nikas DC, Bello L, Zhang J, Kraeft SK, Seyfried NT, Abe T, Chen LB, Carroll RS, Black PM (2000). Expression of cyclooxygenase 2 (COX-2) in human glioma and *in vitro* inhibition by a specific COX-2 inhibitor, NS-398. Cancer Res.

[43] Shono T, Tofilon PJ, Bruner JM, Owolabi O, Lang FF (2001). Cyclooxygenase-2 expression in human gliomas: prognostic significance and molecular correlations. Cancer Res.

[44] Sareddy GR, Geeviman K, Ramulu C, Babu PP (2012). The nonsteroidal anti-inflammatory drug celecoxib suppresses the growth and induces apoptosis of human glioblastoma cells via the NF-kappaB pathway. J Neurooncol.

[45] Zhen C, Chen L, Zhao Q, Liang B, Gu YX, Bai ZF, Wang K, Xu X, Han QY, Fang DF, Wang SX, Zhou T, Xia Q (2012). Gankyrin promotes breast cancer cell metastasis by regulating Rac1 activity. Oncogene.

[46] Sue Ng S, Mahmoudi T, Li VS, Hatzis P, Boersema PJ, Mohammed S, Heck AJ, Clevers H (2010). MAP3K1 functionally interacts with Axin1 in the canonical Wnt signalling pathway. Biol Chem.

