# Supplementary material for: MAGI3 negatively regulates Wnt/β-catenin signaling and suppresses malignant phenotypes of glioma cells
Source: Oncotarget. 2015 Oct 6;6(34):35851–65. doi: 10.18632/oncotarget.5323 (PMC4742146; doi:10.18632/oncotarget.5323)
Supplement: Supplementary file 1 [file oncotarget-06-35851-s001.pdf]

SUPPLEMENTARY FIGURES

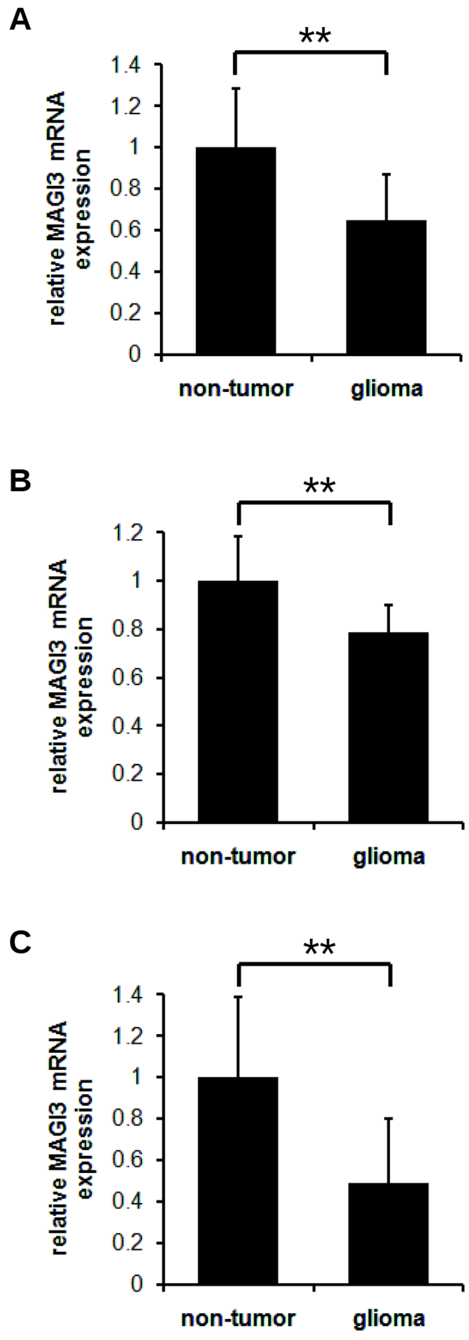

Supplementary Figure S1: MAGI3 expression is downregulated in glioma. MAGI3 gene expression in glioblastomas and non-tumor cases was analyzed based on GSE4290 **A.** GSE7696 **B.** and GSE50161 **C.** datasets. The standardized expression of MAGI3 mRNA in non-tumor tissues and glioblastomas was 1.00 ± 0.28 and 0.65 ± 0.23 for GSE4290, 1.00 ± 0.18 and 0.78 ± 0.12 for GSE7696, 1.00 ± 0.39 and 0.49 ± 0.31 for GSE50161, respectively. The difference between the non-tumor brain group and the high-grade glioma group was statistically significant ($P < 0.01$).

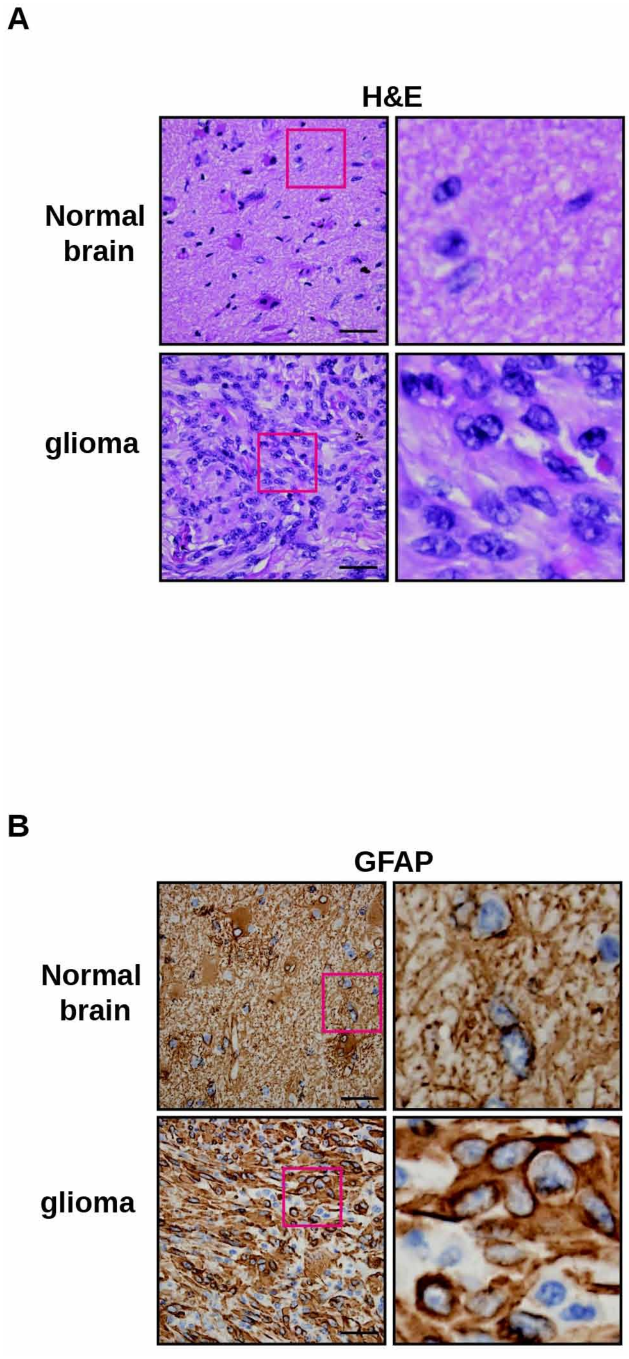

Supplementary Figure S2: GFAP is expressed in both glial cells and glioma cells. Representative images from H&E **A.** or GFAP immunohistochemical staining **B.** on normal brain tissues and glioma samples. The boxed areas in the left images are magnified in the right images. Scale bar, 40 µm. H&E, hematoxylin and eosin.

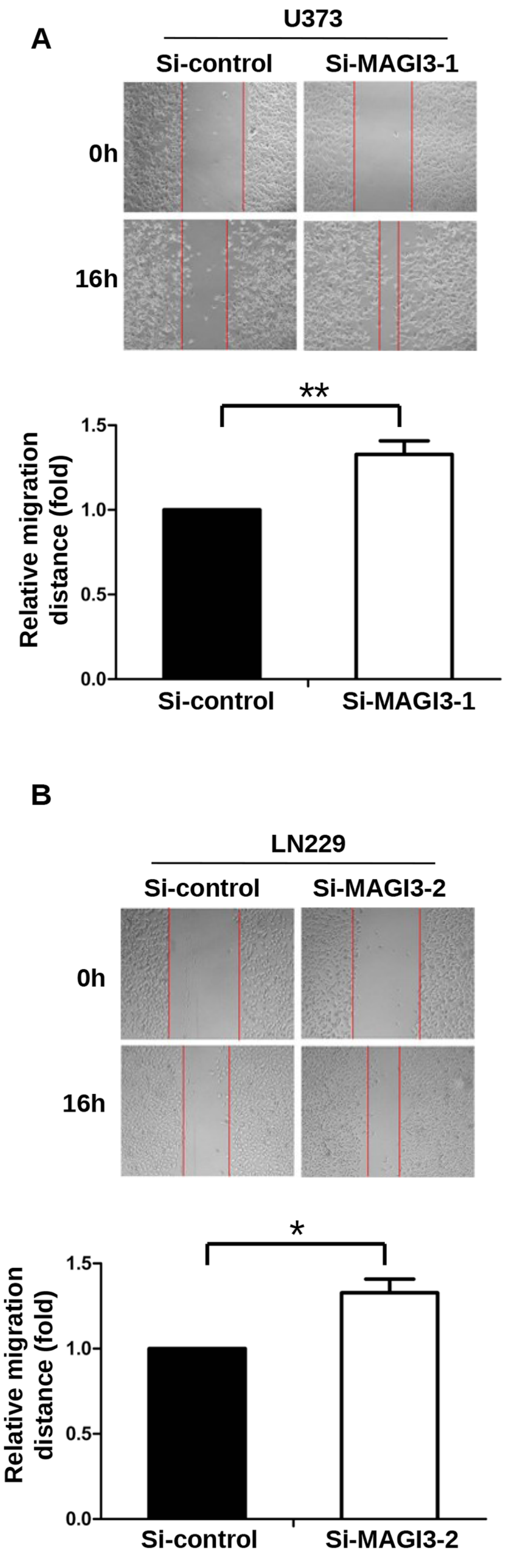

Supplementary Figure S3: Knockdown of MAGI3 expression in glioma cells promotes cell migration. Scratch wounds were created in monolayers of scrambled control or MAGI3 siRNA-transfected U373 **A.** and LN229 **B.** cells at 0 and 16 h of culture. The relative migration distance was quantified (bottom panel). Data represent the mean ± SD of 3 individual experiments. $*P < 0.05$, $**P < 0.01$.

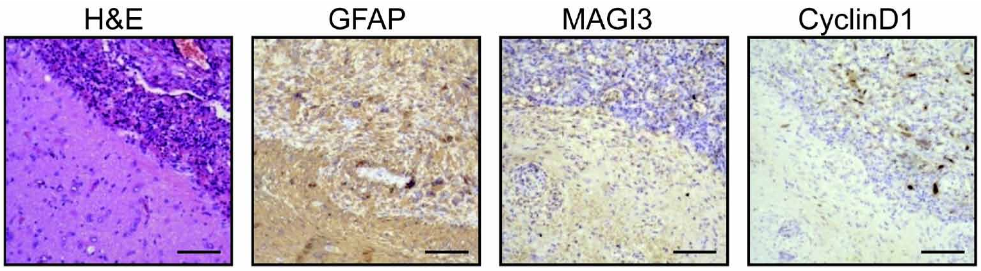

Supplementary Figure S4: MAGI3 protein expression is downregulated concomitant with increased Cyclin D1 expression level in glioma. Representative images from immunohistochemical staining of GFAP, MAGI3 and Cyclin D1 on serial section of glioma. Scale bar, 500 µm. H&E, hematoxylin and eosin.

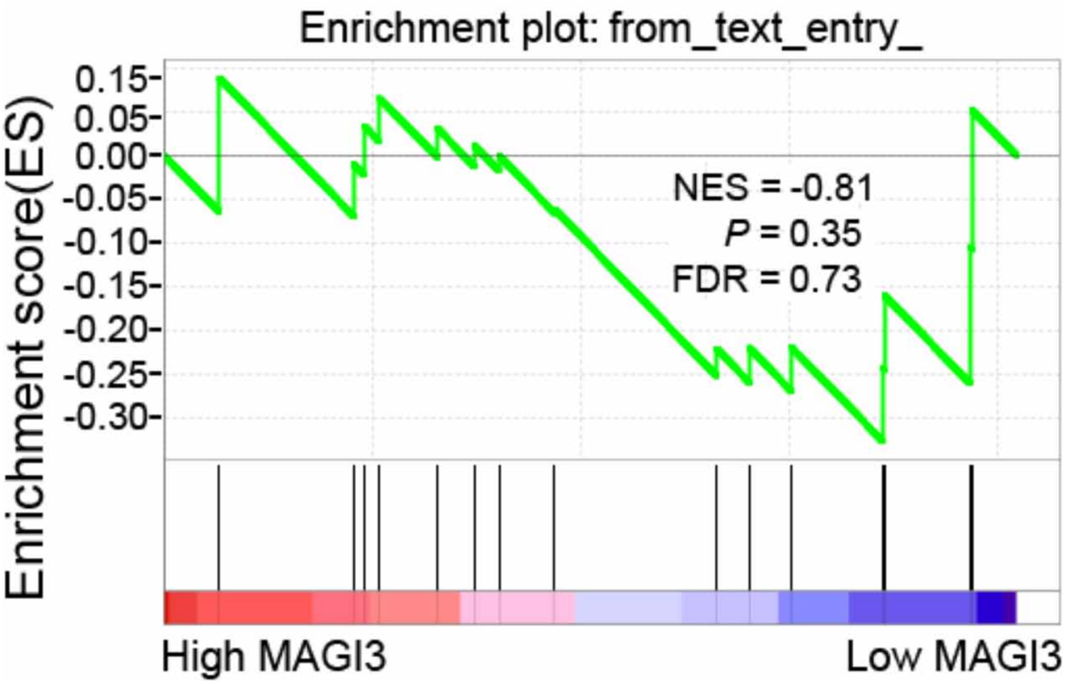

Supplementary Figure S5: MAGI3 expression level is not involved in JNK signaling activation in glioma. Identification of the JNK signaling pathway as a functional target of MAGI3 by Gene set enrichment analysis (GSEA) using a glioma dataset from the GEO profile (GSE4412), which contains 54 grade IV glioma cases. No significant differences in the expression of JNK gene sets was found between high-MAGI3 (MAGI3 expression ≥ median) and low-MAGI3 (MAGI3 expression < median) glioma groups (FDR = 0.73).

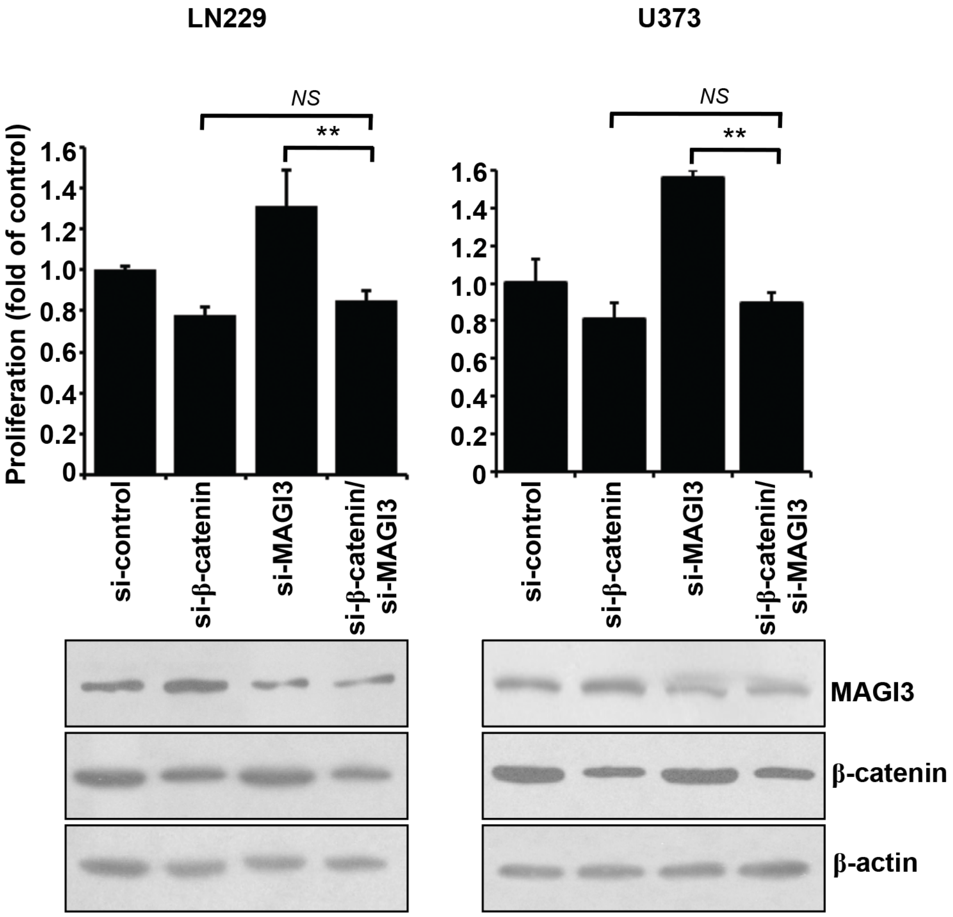

Supplementary Figure S6: MAGI3 inhibits cell proliferation via suppression of β-catenin function. U373 and LN229 cells were transfected with control siRNA, MAGI3 siRNA and/or β-catenin siRNA, and cell proliferation was assessed after 96 h. Values are expressed relative to the absorbance in control siRNA-transfected cells. Error bars represent SD. $**P < 0.01$. NS, no significance.

Supplementary Table S1: Primer used in PCR

Gene amplified/primer	Sequence 5′ - 3′
**β-catenin**	
sense	AAGCTTCCAGACACGCTATCATGC
antisense	ACCAGCTAAACGCACTGCCATT
**MAGI3**	
sense	TCGGTCACACTCATGCAGATGTTG
antisense	TGGTCCATTGAGACCATCACCAGT
**CyclinD1**	
sense	AAGCTGTGCATCTACACCGACAAC
antisense	CATGGAGGGCGGATTGGAAATGAA
**Axin2**	
sense	CCGACTTCAAGTGCAAACTTTCGC
antisense	GCTGTTTCTTACTGCCCACACGAT
**c-Myc**	
sense	CTGGAAGAAATTCGAGCTGA
antisense	ACATACA GTCCTGGATGATGA
**β-actin**	
sense	GTACCACAGGCATTGTGATGGACT
antisense	CTTTGATGTCACGCACGATTTCCC
